# Force of Growing Plants

**Published:** 1887-01

**Authors:** 


### Force of Growing Plants.

The force exerted by growing plants is very great. Fungi are composed of soft tissues, yet a growing mushroon has been known to lift a large paving stone. The rootlets of pines and cedars growing on the sides of rocky declivites penetrate narrow crevices in the rocks, and finally by their growth loosen huge masses and send them tumbling down the cliff. Years ago President Clarke, of the Massachusetts Agricultural College, put a pumpkin into harness and demonstrated that it was capable of lifting thousands of pounds. In a cemetery in Hanover a seed germinated in a crevice beside a tombstone which contained twenty cubic feet. The seedling, now a small tree, has lifted the stone over five inches. Not the least wonderful of phenomena of this class is the force exerted by the radicle of the germinating plant. Darwin has demonstrated that it exerts a force, which in proportion to its size, is astonishing. This force the plantlet utilizes in sending its root into the soil, and the strangest part of strange phenomenon is that the little soft radicle is capable of penetrating soil very much harder than itself.

